# Magnetic Seizure Therapy vs Modified Electroconvulsive Therapy in Patients With Bipolar Mania: A Randomized Clinical Trial

**DOI:** 10.1001/jamanetworkopen.2024.7919

**Published:** 2024-04-29

**Authors:** Shan Chen, Jianhua Sheng, Fuzhong Yang, Yi Qiao, Wenzheng Wang, Hui Wen, Qiao Yang, Xiaochen Chen, Yingying Tang

**Affiliations:** 1 Department of Psychiatry, Shanghai Mental Health Center, Shanghai Jiao Tong University School of Medicine, Shanghai, China; 2 Shanghai Xuhui Mental Health Center, Shanghai, China; 3 Clinical Research Institute, Shanghai Jiao Tong University School of Medicine, Shanghai, China; 4 Shanghai Key Laboratory of Psychotic Disorders, Shanghai Mental Health Center, Shanghai Jiao Tong University School of Medicine, Shanghai, China; 5 Neuroimaging Core, Shanghai Mental Health Center, Shanghai Jiao Tong University School of Medicine, Shanghai, China

## Abstract

**Importance:**

Bipolar mania is a common disabling illness. Electroconvulsive therapy (ECT) is an effective treatment for patients with severe mania, though it is limited by the risk of cognitive adverse effects. Magnetic seizure therapy (MST) as an alternative treatment to ECT for bipolar mania has not yet been reported.

**Objective:**

To compare the effectiveness and cognitive adverse effects of MST and ECT in bipolar mania.

**Design, Setting, and Participants:**

This randomized clinical trial was conducted at the Shanghai Mental Health Center from July 1, 2017, through April 26, 2021. Forty-eight patients with bipolar mania were recruited and randomly allocated to receive MST or ECT. The data analysis was performed from June 5, 2021, through August 30, 2023.

**Interventions:**

Patients completed 2 or 3 sessions of MST or ECT per week for a total of 8 to 10 sessions. The MST was delivered at 100% device output with a frequency of 75 Hz over the vertex.

**Main Outcomes and Measures:**

The primary outcomes were reduction of total Young Manic Rating Scale (YMRS) score and response rate (more than 50% reduction of the total YMRS score compared with baseline). An intention-to-treat (ITT) analysis and repeated-measures analyses of variance were conducted for the primary outcomes.

**Results:**

Twenty patients in the ECT group (mean [SD] age, 31.6 [8.6] years; 12 male [60.0%]) and 22 patients in the MST group (mean [SD] age, 34.8 [9.8] years; 15 male [68.2%]) were included in the ITT analysis. The response rates were 95.0% (95% CI, 85.4%-100%) in the ECT group and 86.4% (95% CI, 72.1%-100%) in the MST group. The YMRS reduction rate (*z* = −0.82; 95% CI, −0.05 to 0.10; *P* = .41) and response rate (χ^2^ = 0.18; 95% CI, −0.13 to 0.31; *P* = .67) were not significantly different between the groups. The time-by-group interaction was significant for the language domain (*F*_1,24_ = 7.17; *P* = .01), which was well preserved in patients receiving MST but worsened in patients receiving ECT. No serious adverse effects were reported in either group.

**Conclusions and Relevance:**

These findings suggest that MST is associated with a high response rate and fewer cognitive impairments in bipolar mania and that it might be an alternative therapy for the treatment of bipolar mania.

**Trial Registration:**

ClinicalTrials.gov Identifier: NCT03160664

## Key Points

**Question:**

Does magnetic seizure therapy (MST) have comparable efficacy to modified electroconvulsive therapy (ECT) for bipolar mania?

**Findings:**

This randomized clinical trial of 22 patients treated with MST and 20 treated with ECT found good response rates (86.4% and 95.0%, respectively). Language function was well preserved in patients receiving MST, though it was worsened in patients receiving ECT.

**Meaning:**

These findings show that MST may be an alternative treatment for bipolar mania with fewer effects on patients’ language ability.

## Teaser

This randomized clinical trial compares the effectiveness and cognitive adverse effects of magnetic seizure therapy and electroconvulsive therapy among adults with bipolar mania in China.

## Introduction

Bipolar mania is a common and disabling mental illness characterized by a persistently elevated or irritable mood and abnormally increased activity or energy.^1^ These symptoms may lead to functional impairment, hospitalization, and an increased risk of suicide.^2,3^ First-line pharmacologic monotherapy, including lithium, divalproex, and other anticonvulsants, effectively improves acute manic symptoms in approximately 50% of patients with bipolar mania.^1,3,4^ Electroconvulsive therapy (ECT) is a second-line treatment for patients with mania who have an inadequate response to first-line monotherapy.^1,5^ The largest study to date suggested an 84.4% response rate to ECT for mania in a Swedish population.^6^ The use of ECT to treat bipolar mania is limited by its potential adverse effects, such as cognitive impairment and stigma surrounding the treatment. Therefore, novel treatments that can rapidly and remarkably improve symptoms and reduce cognitive side effects are needed for patients with mania.

A candidate treatment is magnetic seizure therapy (MST), which has been shown to be effective and safe in treatment-resistant depression.^7,8^ It uses magnetic pulses rather than electrical currents in patients under general anesthesia.^9^ The advantage of magnetic pulses is that they are not impeded by the skull and can be targeted over the cortical region to avoid or minimize cognitive adverse effects.^10^ Magnetic seizure therapy has a robust antidepressant effect,^7,8,9,10,11,12^ which is indistinguishable from that of ECT as reported by a recent randomized clinical trial (RCT).^13^ Magnetic seizure therapy may also improve psychotic symptoms in patients with schizophrenia.^14^ Preliminary evidence has shown that patients receiving MST may have shorter recovery and reorientation times and less cognitive impairment than those receiving ECT.^7,13,14^ However, the effects of MST on acute manic symptoms have yet to be investigated.

In this study, we compare the effectiveness of MST and ECT for the treatment of bipolar mania. We hypothesized that the response rate would be comparable between MST and ECT but that cognitive functions would be better preserved by MST. We also examined the safety and tolerability of MST.

## Methods

### Participants

The experimental protocol for this randomized clinical trial (Supplement 1) was approved by the ethics committee of the Shanghai Mental Health Center (SMHC) and registered at ClinicalTrials.gov (NCT03160664). The study followed the Consolidated Standards of Reporting Trials (CONSORT) reporting guideline. The sample size calculation is described in eMethods 1 in Supplement 2. Written informed consent was obtained from the patients or their legal guardians.

From July 1, 2017, through April 26, 2021, inpatients with manic episodes of bipolar disorder at SMHC who met the diagnostic criteria according to the fifth edition of the *Diagnostic and Statistical Manual of Mental Disorders*^15^ were screened and included. Patients aged 18 to 55 years with moderate to severe manic symptoms, Young Manic Rating Scale (YMRS) scores of 10 or higher, and clinical indications for convulsive treatment were included in the study. Individuals with additional mental disorders according to the *Diagnostic and Statistical Manual of Mental Disorders*, alcohol or substance dependence within 6 months prior to the trial, severe physical diseases (eg, stroke, heart failure, liver failure, neoplasm, immune deficiency), abnormal laboratory findings that could affect efficacy or safety, failure to respond to an adequate ECT trial, pregnancy or plans to become pregnant during the study period, unremovable metal implants, or other conditions inappropriate for participation were excluded.

### MST and ECT Procedures

#### Randomization and Masking

**Figure 1.**
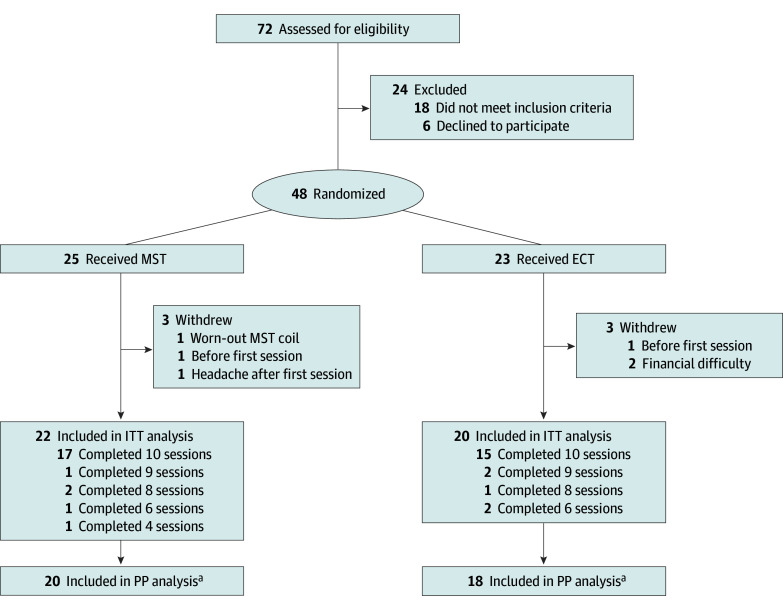
CONSORT Diagram ECT indicates electroconvulsive therapy; ITT, intention to treat; MST, magnetic seizure therapy; PP, per protocol. ^a^Completing at least 8 sessions.

A sequence of random numbers corresponding to patient serial numbers was generated using SPSS, version 22 (IBM Corporation). Each random number was assigned to the MST or ECT group. Forty-eight patients were randomly assigned a number in 1 of the groups (Figure 1). Regular medication was not restricted during the study. The staff members who performed the clinical and neurocognitive assessments were masked to the randomization during the whole procedure. The MST or ECT operators were aware of the treatment. All procedures and room setups prior to treatment were the same for the MST and ECT groups to ensure that patients were masked to the group information.

#### Anesthesia

General anesthesia was administered to each patient at the beginning of each ECT or MST session using propofol (1.82-2.44 mg/kg) and etomidate (0.21-0.3 mg/kg). Succinylcholine (1 mg/kg) was used for muscle relaxation to prevent fractures and other injuries, and atropine (0.5 mg) was administered to reduce respiratory secretions. After each ECT or MST session, the patients remained under observation for 30 minutes and were not permitted to eat for 2 hours.

#### MST Procedure

Patients completed 2 or 3 sessions of MST per week (on alternating days), totaling 8 to 10 sessions within 4 weeks. The MST was administered using a MagPro X100 device with a twin coil (Twin Coil-XS; MagVenture A/S) centered at the vertex and with maximum device output intensity at a frequency of 75 Hz. The duration of magnetic stimulations was determined using a titration method until a proper seizure (seizure duration of 15 seconds or longer, monitored by electroencephalogram [EEG]) was generated. The duration was initiated at 4 seconds and increased by 4 seconds or 8 seconds up to a maximum of 20 seconds.

#### ECT Procedure

Patients completed 2 or 3 sessions of ECT per week (on alternating days), totaling 8 to 10 sessions within 4 weeks. Bitemporal ECT was performed using a Thymatron System IV device (Somatics, LLC) according to routine clinical practice at SMHC. The electrical stimulation wave width was 1.0 ms. The energy of electrical stimulation was determined according to the patients’ age (age × 0.8 × 100%) and increased by 5% until a proper seizure (seizure duration of 25 seconds or longer) was achieved.

#### EEG Recording

During each MST and ECT session, EEG patch electrodes were placed in the bilateral frontal regions. The epileptiform discharge times induced by MST or ECT were recorded as the effective seizure durations.

### Outcome Measures

Designated, qualified psychiatrists (W.W. and H.W.) assessed the patients’ manic symptoms using the YMRS^16^ before and after MST and ECT treatments. A reduction in the total YMRS score was used as the primary outcome. A response to treatment was defined as a greater than 50% reduction in the total YMRS score compared with baseline.

We assessed the patients’ depressive symptoms using the Montgomery-Åsberg Depression Rating Scale (MADRS) and neurocognitive function using the Chinese version of the Repeatable Battery for the Assessment of Neuropsychological Status (RBANS)^17^ before and after the treatments. The RBANS has good reliability and validity^18^ and includes 12 tasks for 5 cognitive domains (immediate memory, visuospatial/constructional, language, attention, and delayed memory). A trained staff member (S.C.) conducted the RBANS. Some patients did not complete the RBANS due to emotional instability and uncooperative behavior during the acute phase. The total MADRS and RBANS scores and RBANS cognitive domain scores were secondary outcomes.

### Statistical Analysis

The data analysis was performed from June 5, 2021, through August 30, 2023. Patients who completed at least 1 MST or ECT treatment session were included in the intention-to-treat (ITT) analysis. The value of the last assessment was the final efficacy indicator.^19^ The per-protocol analysis and findings are included in eMethods 2 and eTables 1 to 3 in Supplement 2.

Between-group comparisons for demographic and clinical characteristics were performed using independent *t* tests for continuous variables with normal distributions and Mann-Whitney *U* tests for data not normally distributed. A repeated-measures analyses of variance was performed with a within-group factor of time (before vs after treatment) and a between-group factor of group (MST vs ECT) to assess the primary and secondary outcomes.

For neurocognitive assessments, we added age, sex, and years of education as covariates. The Bonferroni method was used for adjusting the multiple comparisons. The correlations between seizure duration and changes in total YMRS scores and the language and attention domain scores of the RBANS were analyzed using Pearson or Spearman correlation tests according to the distributions of the data. All statistical analyses were performed using SPSS, version 22. Statistical significance was set at 2-sided *P* < .05.

## Results

### Demographic and Clinical Characteristics

Twenty patients in the ECT group (mean [SD] age, 31.6 [8.6] years; 8 female [40.0%]; 12 male [60.0%]) and 22 patients in the MST group (mean [SD] age, 34.8 [9.8] years; 7 female [31.8%]; 15 male [68.2%]) were included in the ITT analysis (Figure 1). Table 1 presents the patient demographic and clinical characteristics. The age, sex, years of education, marital status, total YMRS scores, YMRS item scores, and total MADRS scores at baseline did not differ between the 2 groups. At baseline, the daily chlorpromazine-equivalent doses of atypical antipsychotics in the MST group were significantly higher than those in the ECT group (*z* = −2.457; *P* = .02). After treatment, the daily chlorpromazine-equivalent doses of atypical antipsychotics were comparable between the MST and ECT groups (*z* = −0.183; *P* = .86). The increase in daily chlorpromazine-equivalent doses was significantly more in the ECT group than in the MST group (*z* = −2.356; *P* = .02).

**Table 1.** Demographic and Clinical Characteristics of Patients Before Receiving Modified ECT and MST

Characteristic	ECT group (n = 20)	MST group (n = 22)
Age, mean (SD), y	31.6 (8.6)	34.8 (9.8)
Sex, No. (%)		
Female	8 (40.0)	7 (31.8)
Male	12 (60.0)	15 (68.2)
Education, median (IQR), y	16.0 (15.3-16.0)	16.0 (9.0-16.0)
Marriage status, No. (%)		
Single	12 (60.0)	13 (59.1)
Married	8 (40.0)	9 (40.9)
Disease duration, median (IQR), mo	61.5 (31.0-156.0)	162.0 (63.0-210.0)
Family history of mental disorders, No. (%)		
Yes	8 (40.0)	3 (13.6)
No	12 (60.0)	19 (86.4)
Daily chlorpromazine-equivalent doses, median (IQR), mg	350.0 (208.3-504.2)	516.7 (400.0-800.0)
Use of mood stabilizers, No. (%)		
Yes	15 (75.0)	15 (68.2)
No	5 (25.0)	7 (31.8)
Use of benzodiazepines, No. (%)		
Yes	4 (20.0)	4 (18.1)
No	16 (80.0)	18 (81.9)
YMRS total score, median (IQR), points^a^	34.5 (29.3-39.8)	36.0 (30.8-43.0)
Elevated mood, mean (SD)	3.5 (0.5)	3.5 (0.6)
Increased motor activity or energy, median (IQR)	3.0 (3.0-4.0)	3.5 (3.0-4.0)
Sexual interest, median (IQR)	1.5 (1.0-3.0)	2.0 (1.0-3.3)
Sleep, median (IQR)	3.0 (3.0-3.0)	3.0 (3.0-3.0)
Irritability, median (IQR)	4.5 (4.0-6.8)	5.0 (3.8-6.0)
Speech rate and amount increase, median (IQR)	5.0 (5.0-6.0)	5.0 (4.0-6.3)
Language-thought disorder, median (IQR)	3.0 (2.0-3.8)	3.0 (2.0-3.0)
Content of thinking, median (IQR)	4.5 (3.0-6.0)	4.0 (1.4-6.0)
Disruptive or aggressive behavior, mean (SD)	3.4 (2.2)	3.6 (2.3)
Appearance, median (IQR)	2.0 (1.0-2.0)	2.0 (0.8-3.0)
Insight, median (IQR)	2.5 (0-3.0)	3.0 (1.8-3.3)
MADRS score, median (IQR), points^b^	6.5 (3.0-11.8)	4.5 (1.5-8.0)

Abbreviations: ECT, electroconvulsive therapy; MADRS, Montgomery-Åsberg Depression Rating Scale; MST, magnetic seizure therapy; YMRS, Young Manic Rating Scale.

^a^ Scores range from 0 to 60, with higher scores indicating more symptoms of mania.

^b^ Scores range from 0 to 60, with higher scores indicate more symptoms of depression.

### Clinical Outcomes

Nineteen of 20 patients (95.0%; 95% CI, 85.4%-100%) responded to ECT, and 19 of 22 patients (86.4%; 95% CI, 72.1%-100%) responded to MST (χ^2^ = 0.18; 95% CI, −0.13 to 0.31; *P* = .67). The reduction of the total YMRS scores (mean [SD]: ECT, 29.75 [10.1]; MST, 28.73 [11.7]; *t* = −0.30; Cohen *d* = 0.09; 95% CI, −5.81 to 7.85; *P* = .76) and the YMRS reduction rate (mean [SD]: ECT, 0.83 [0.17]; MST, 0.78 [0.22]; *z* = −0.82; Cohen *d* = 0.24; 95% CI, −0.05 to 0.10; *P* = .41) were not significantly different between the groups.

The main time effects on the total YMRS scores (*F*_1,40_ = 299.5; η^2^ = 0.88; *P* < .001) and total MADRS scores (*F*_1,40_ = 51.1; η^2^ = 0.56; *P* < .001) were significant. The 11 YMRS item scores (Table 2) were also significant, and all survived Bonferroni correction for multiple comparisons except the insight item score. No significant group effects or time-by-group interactions for the total YMRS or item scores were identified.

**Table 2.** Clinical Outcomes Before and After Receiving ECT and MST

Clinical measure	Score, mean (SD), points				Time effect			Group effect			Time × group interaction		
	ECT group (n = 20)		MST group (n = 22)										
	Before	After	Before	After	*F*_1,40_	*P* value	η^2^	*F*_1,40_	*P* value	η^2^	*F*_1,40_	*P* value	η^2^
YMRS	35.4 (7.9)	4.6 (4.3)	36.3 (8.1)	8.2 (9.3)	299.5	<.001^a^	0.88	0.81	.37	0.02	0.09	.76	<0.01
Elevated mood	3.5 (0.5)	0.8 (0.7)	3.5 (0.6)	1.0 (1.0)	357.4	<.001^a^	0.90	0.31	.58	0.01	0.53	.47	0.01
Increased motor activity or energy	3.3 (0.6)	0.7 (0.7)	3.5 (0.5)	0.9 (1.0)	281.2	<.001^a^	0.88	1.63	.21	0.04	0.002	.97	<0.01
Sexual interest	1.9 (1.4)	0.3 (0.7)	2.2 (1.2)	0.3 (0.7)	79.9	<.001^a^	0.67	1.09	.48	0.01	0.49	.44	0.02
Sleep	2.9 (0.4)	0.4 (0.5)	3.0 (0.6)	0.4 (0.7)	412.1	<.001^a^	0.91	0.24	.63	0.01	<0.001	.99	<0.01
Irritability	4.8 (2.0)	0.4 (0.7)	4.7 (2.0)	0.7 (1.1)	180.1	<.001^a^	0.82	0.12	.73	<0.01	0.41	.53	0.01
Speech rate amount increase	5.4 (1.1)	0.7 (0.9)	5.1 (1.6)	1.0 (1.3)	270.5	<.001^a^	0.87	0.02	.88	<0.01	1.29	.26	0.03
Language-thought disorder	2.6 (1.2)	0.4 (0.6)	2.6 (1.1)	0.4 (0.9)	136.4	<.001^a^	0.77	0.01	.92	<0.01	0.03	.87	<0.01
Content of thinking	4.2 (2.0)	0.3 (0.6)	3.9 (2.7)	0.7 (1.4)	90.3	<.001^a^	0.69	0.01	.92	<0.01	0.81	.38	0.02
Disruptive or aggressive behavior	3.4 (2.2)	0.1 (0.3)	3.6 (2.3)	0.4 (1.1)	85.8	<.001^a^	0.68	0.34	.56	0.01	0.01	.92	<0.01
Appearance	1.6 (0.9)	0.3 (0.6)	1.8 (1.3)	0.2 (0.5)	61.0	<.001^a^	0.60	0.06	.81	<0.01	0.45	.50	0.01
Insight	2.0 (1.5)	1.5 (1.1)	2.5 (1.4)	1.7 (1.1)	5.4	.03^b^	0.12	1.52	.23	0.04	0.32	.58	0.01
MADRS	8.8 (8.4)	1.8 (3.1)	5.4 (4.6)	1.1 (2.8)	51.1	<.001^a^	0.56	2.14	.15	0.05	3.20	.08	0.07

Abbreviations: ECT, electroconvulsive therapy; MADRS, Montgomery-Åsberg Depression Rating Scale; MST, magnetic seizure therapy; YMRS, Young Manic Rating Scale.

^a^ *P* < .001, which survived after Bonferroni correction for multiple comparisons.

^b^ *P* < .05, which did not survive after Bonferroni correction for multiple comparisons.

### Neurocognitive Outcomes

Fourteen patients in the ECT group and 15 in the MST group completed the RBANS neurocognitive assessments before and after treatment. No significant time effects (*F*_1,24_ = 0.72; η^2^ = 0.03; *P* = .41), group effects (*F*_1,24_ = 1.33; η^2^ = 0.05; *P* = .26), or time-by-group interactions (*F*_1,24_ = 0.29; η^2^ = 0.01; *P* = .60) for the total RBANS scores were identified (Table 3).

**Table 3.** Neurocognitive Outcomes Before and After Receiving ECT and MST

Neurocognitive measure	Score, mean (SD), points				Time effect			Group effect				Time × group interaction		
	ECT group (n = 14)		MST group (n = 15)											
	Before	After	Before	After	*F*_1,24_	*P* value	η^2^	*F*_1,24_	*P* value	η^2^	*F*_1,24_		*P* value	η^2^
RBANS	78.8 (16.7)	78.4 (13.9)	82.2 (13.4)	85.3 (14.0)	0.72	.41	0.03	1.33	.26	0.05	0.29		.60	0.01
Immediate memory	77.6 (12.4)	82.1 (19.3)	79.5 (13.4)	81.3 (16.0)	0.16	.69	0.01	0.06	.81	<0.01	0.10		.76	<0.01
Visuospatial/ constructional	88.2 (15.0)	94.2 (11.9)	91.8 (18.4)	92.1 (19.8)	0.04	.84	<0.01	0.07	.80	<0.01	0.73		.40	0.03
Language	87.4 (16.4)	77.5 (12.9)	90.8 (11.1)	95.3 (13.7)	0.66	.43	0.03	6.44	.02	0.21	7.17		.01^a^	0.23
Attention	86.5 (19.2)	92.8 (14.7)	91.7 (17.4)	97.1 (16.6)	3.35	.08	0.12	1.08	.32	0.04	0.05		.83	<0.01
Delayed memory	78.1 (19.6)	70.4 (17.9)	78.9 (19.8)	78.9 (17.8)	1.08	.31	0.04	0.44	.51	0.02	0.48		.50	0.02

Abbreviations: ECT, electroconvulsive therapy; MST, magnetic seizure therapy; RBANS, Repeatable Battery for the Assessment of Neuropsychological Status.

^a^ The significant time-by-group interaction survived Bonferroni correction.

For the language domain, a significant time-by-group interaction (*F*_1,24_ = 7.17; η^2^ = 0.23; *P* = .01) and group effect (*F*_1,24_ = 6.44; η^2^ = 0.21; *P* = .02) were observed. The significant time-by-group interaction survived Bonferroni correction. Patients receiving ECT had worse language performance after treatment (*z* = −2.00; 95% CI, 0-20.5; *P* = .045), while patients receiving MST had no significant changes in language performance after treatment (*z* = −1.32; 95% CI, −11.5 to 2.5; *P* = .19).

**Figure 2.**
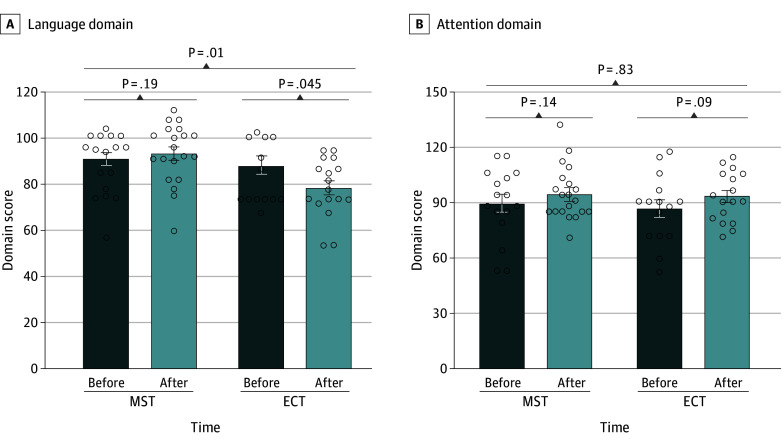
Time-by-Group Interaction on Language Performance and Time Effect on Attention Performance in Patients With Mania Undergoing Magnetic Seizure Therapy (MST) and Electroconvulsive Therapy (ECT) Dots indicate individual patients, bars indicate the mean, and whiskers indicate the standard error of the mean.

No significant time effect was observed for the attention domain (*F*_1,24_ = 3.35; *P* = .08; η^2^ = 0.12). Patients in the ECT group (*t* = 1.86; 95% CI, −1.0 to 13.6; *P* = .09) had subtle improvement in the attention scores after treatment, whereas the attention scores of those in the MST group were not significantly different before and after treatment (*t* = −1.59; 95% CI, −1.9 to 12.9; *P* = .14) (Figure 2). No significant time effects, group effects, or time-by-group interactions were observed for the immediate memory, visuospatial/constructional, or delayed memory domains of the RBANS.

### EEG Seizure Durations

The mean (SD) seizure duration in the ECT group (37.32 [9.8] seconds) was significantly longer than that in the MST group (10.96 [3.5] seconds; *z* = −5.48; 95% CI, 21.1-30.6; *P* < .001). The seizure duration was negatively correlated with changes in language scores in the MST group (*r* = −0.67; 95% CI, −0.91 to −0.35; *P* = .009).

### Safety, Tolerability, and Adverse Effects

No serious adverse effects were reported in either group. Overall, 20 patients (80.0%) and 18 patients (78.3%) in the MST and ECT groups, respectively, completed at least 8 treatment sessions. Five patients (2 in the MST group and 3 in the ECT group) reported transient general anesthesia reactions, such as headache and nausea, which were soon relieved after treatment. One patient reported general discomfort after 6 MST sessions and did not comply with MST and pharmaceutical treatments. In the MST group, 3 patients in the MST group withdrew early due to a headache (n = 1), a worn-out MST coil (n = 1), and stigma (n = 1). In the ECT group, 3 patients withdrew early due to financial difficulties in hospitalization (n = 2) and stigma (n = 1).

## Discussion

To our knowledge, this study is the first to examine MST effects on acute manic symptoms. Patients showed a response rate of 86.4% after 8 to 10 sessions of MST, which did not significantly differ from a response rate of 95.0% after ECT. The language function was well preserved after receiving MST, though it worsened after receiving ECT. Magnetic seizure therapy induced a shorter seizure duration than ECT and negatively correlated with language score changes. These preliminary findings suggest that MST may be an alternative treatment for bipolar mania.

This study included patients with severe manic symptoms for whom ECT was indicated. The good antimanic effect of MST did not differ significantly from ECT. Improvements in manic symptoms after ECT were consistent with those reported in previous studies.^2,5,6^ The YMRS items also showed significant reductions in patients receiving MST, including elevated mood, increased motor activity or energy, irritability, and disruptive or aggressive behaviors. The preliminary antimanic efficacy of MST encourages performance of future RCTs with larger sample sizes.

Magnetic seizure therapy is typically used to treat treatment-resistant depression,^11,13,20,21^ and few attempts have been made to treat bipolar depression.^22,23,24,25^ The antidepressant effects of MST were not significantly different from those of ECT.^11,20,21,26^ Tang and colleagues^24,25^ conducted an open-label MST study regarding treatment-resistant bipolar depression and found that depressive symptoms and suicidal ideation significantly improved with minimal cognitive effects. Noda et al^27^ reported that 2 patients with major depressive disorder (MDD) developed manic symptoms after 6 and 23 MST sessions. Thus, MST may be effective in patients with various states of bipolar depression and mania, though more research is necessary to elucidate these effects.

Some preliminary evidence shows that MST may minimize cognitive adverse effects compared with ECT. No global cognitive impairments were observed in either group after treatment in this study. Two significant cognitive effects were noted: language function was well preserved in patients undergoing MST, though it was worsened in patients undergoing ECT, and the attention of patients in both groups showed improvement after treatment.

The different effects on language function between MST and ECT are consistent with previous studies regarding MDD and schizophrenia.^14,28,29^ Wang et al^28^ reported some language improvement after accelerated MST in patients with MDD. Jiang et al^14^ observed significant group and time effects on language function in patients with schizophrenia. The findings from a meta-analysis on depression suggested that MST may affect language fluency, immediate and delayed word recall, and immediate and delayed visuospatial recall less than ECT.^7^

The time effect for the attention domain showed slightly increased scores after ECT and MST but was not statistically significant. Patients with mania are characterized by distractibility, as their attention is easily drawn to unimportant or irrelevant stimuli. Improved attentional performance may be attributed to improved manic symptoms and more stable overall states in the study patients after treatment.

A recent neuroimaging review suggested that the functional neuroanatomy of mania may include hypoactivity in the right ventral prefrontal cortex and hyperactivity in the left amygdala, anterior cingulate cortex, and basal ganglia.^30^ Magnetic seizure therapy could induce metabolic changes in the bilateral frontal cortex and left striatum in patients with treatment-resistant depression.^11^ The different effects on cognition between MST and ECT may be associated with the different diffusion patterns between magnetic and electrical stimulation. The scalp and skull shunt the electrical current of ECT, leading to a nonfocal and widespread electrical charge to the brain.^10,31^ Magnetic seizure therapy generates a focal electric field that is more confined to the superficial cortex.^31^ The involvement of deep brain structures (ie, the hippocampus) plays a key role in ECT efficacy and cognitive side effects.^32,33,34^ A previous study reported that ECT induced gray matter volume increases in the bilateral parahippocampal gyrus and hippocampus, right temporal pole, and right insula in patients with schizophrenia, while MST did not.^35^

Overall, both ECT and MST were effective and tolerated by our patients with bipolar mania. Eight treatment sessions were completed by 80.0% of patients in the MST group and 78.3% of patients in the ECT group, with no serious adverse effects in either group.

In this pilot study, we selected an MST frequency of 75 Hz. Previous studies suggested that high-frequency MST (100 Hz) could lead to a better remission rate than low-frequency (25 Hz) and medium-frequency (50 or 60 Hz) MST^10^ but a lower reduction of suicidality.^36^ The target symptoms and population may affect the association of MST frequency and efficacy,^14,37^ which remains unclear. The electric field induced by ECT parameters has been attributed to antidepressant or cognitive outcomes and brain neuroplasticity.^38,39,40^ Future works are needed to optimize MST parameters (ie, frequency, electrode placement, intensity) by including the realistic head model, electric field modeling, and comparisons among different MST frequencies.

### Limitations

This study has several limitations. First, the lack of precise sample size estimation for a noninferiority design and small sample size was a major limitation. Second, we did not restrict the use of concomitant psychotropic medications during ECT or MST. The daily chlorpromazine-equivalent doses were higher in the MST group than in the ECT group at baseline, though they were comparable after treatment. Thus, the effects of pharmacologic therapies cannot be excluded entirely. Third, the study was ceased before a sample size of 60 patients was reached due to the COVID-19 pandemic, which may introduce bias. Fourth, only the efficacy of 75-Hz MST was examined and compared with bitemporal ECT. Unilateral ECT with an ultrabrief pulse may be a better comparator with more cognitively safe and similar efficacy to bitemporal ECT. The MST and ECT parameters must be further optimized. Fifth, the lack of clinical and cognitive measures after each session made it impossible to depict the trajectory of manic symptoms during the 10-session treatments. Deng et al^13^ recently reported that fewer sessions are needed to achieve remission with ECT than MST for depression. Sixth, our study lacked autobiographical memory assessment, a main cognitive domain examined in ECT and MST studies.^10,27^ In summary, our findings are preliminary and need to be validated by future large-scale RCTs regarding optimized MST protocols.

## Conclusions

The findings of this pilot study show that MST and ECT are associated with high response rates in bipolar mania. However, due to the small sample size, clinically significant differences in response rates between these treatments cannot be ruled out. Magnetic seizure therapy induced a shorter seizure duration and had fewer effects on language dysfunction than ECT. These findings suggest that MST is a potential alternative treatment for bipolar mania. Future studies with larger sample sizes, longer follow-up periods, and different MST protocols are needed to validate the efficacy of MST and facilitate its clinical application.

## Notes

Supplement 1.
Trial Protocol

Supplement 2. **eMethods 1.** Sample Size Calculation **eMethods 2.** Results of Per-Protocol Analysis
eReference
**eTable 1.** Demographic and Clinical Characteristics of Patients Before Receiving Modified Electroconvulsive Therapy (ECT) and Magnetic Seizure Therapy (MST) **eTable 2.** Clinical Characteristics of Patients Before and After Receiving Magnetic Seizure Therapy (MST) and Modified Electroconvulsive Therapy (ECT) **eTable 3.** Neurocognitive Assessments of Patients Before and After Receiving Magnetic Seizure Therapy (MST) and Modified Electroconvulsive Therapy (ECT)

Supplement 3.
Data Sharing Statement

## References

[1] Yatham LN, Kennedy SH, Parikh SV, . Canadian Network for Mood and Anxiety Treatments (CANMAT) and International Society for Bipolar Disorders (ISBD) 2018 guidelines for the management of patients with bipolar disorder. Bipolar Disord. 2018;20(2):97-170. doi:10.1111/bdi.12609 29536616 PMC5947163

[2] Mukherjee S, Sackeim HA, Schnur DB. Electroconvulsive therapy of acute manic episodes: a review of 50 years’ experience. Am J Psychiatry. 1994;151(2):169-176. doi:10.1176/ajp.151.2.169 8296883

[3] Mutz J. Brain stimulation treatment for bipolar disorder. Bipolar Disord. 2023;25(1):9-24. doi:10.1111/bdi.13283 36515461 PMC10210071

[4] Kishi T, Ikuta T, Matsuda Y, . Pharmacological treatment for bipolar mania: a systematic review and network meta-analysis of double-blind randomized controlled trials. Mol Psychiatry. 2022;27(2):1136-1144. doi:10.1038/s41380-021-01334-4 34642461 PMC9054678

[5] Small JG, Klapper MH, Kellams JJ, . Electroconvulsive treatment compared with lithium in the management of manic states. Arch Gen Psychiatry. 1988;45(8):727-732. doi:10.1001/archpsyc.1988.01800320037004 2899425

[6] Popiolek K, Bejerot S, Landén M, Nordenskjöld A. Association of clinical and demographic characteristics with response to electroconvulsive therapy in mania. JAMA Netw Open. 2022;5(6):e2218330. doi:10.1001/jamanetworkopen.2022.18330 35737387 PMC9227004

[7] Chen M, Yang X, Liu C, . Comparative efficacy and cognitive function of magnetic seizure therapy vs. electroconvulsive therapy for major depressive disorder: a systematic review and meta-analysis. Transl Psychiatry. 2021;11(1):437. doi:10.1038/s41398-021-01560-y 34420033 PMC8380249

[8] Jiang J, Zhang C, Li C, . Magnetic seizure therapy for treatment-resistant depression. Cochrane Database Syst Rev. 2021;6(6):CD013528. 34131914 10.1002/14651858.CD013528.pub2 PMC8205924

[9] Lisanby SH, Schlaepfer TE, Fisch HU, Sackeim HA. Magnetic seizure therapy of major depression. Arch Gen Psychiatry. 2001;58(3):303-305. doi:10.1001/archpsyc.58.3.303 11231838

[10] Daskalakis ZJ, Dimitrova J, McClintock SM, . Magnetic seizure therapy (MST) for major depressive disorder. Neuropsychopharmacology. 2020;45(2):276-282. 31486777 10.1038/s41386-019-0515-4 PMC6901571

[11] Kayser S, Bewernick BH, Matusch A, Hurlemann R, Soehle M, Schlaepfer TE. Magnetic seizure therapy in treatment-resistant depression: clinical, neuropsychological and metabolic effects. Psychol Med. 2015;45(5):1073-1092. doi:10.1017/S0033291714002244 25420474

[12] Sun Y, Blumberger DM, Mulsant BH, . Magnetic seizure therapy reduces suicidal ideation and produces neuroplasticity in treatment-resistant depression. Transl Psychiatry. 2018;8(1):253. doi:10.1038/s41398-018-0302-8 30470735 PMC6251931

[13] Deng ZD, Luber B, McClintock SM, Weiner RD, Husain MM, Lisanby SH. Clinical outcomes of magnetic seizure therapy vs electroconvulsive therapy for major depressive episode: a randomized clinical trial. JAMA Psychiatry. 2024;81(3):240-249. doi:10.1001/jamapsychiatry.2023.4599 38055283 PMC10701670

[14] Jiang J, Li J, Xu Y, . Magnetic seizure therapy compared to electroconvulsive therapy for schizophrenia: a randomized controlled trial. Front Psychiatry. 2021;12:770647. doi:10.3389/fpsyt.2021.770647 34899429 PMC8656219

[15] American Psychiatric Association. Diagnostic and Statistical Manual of Mental Disorders. 5th ed. American Psychiatric Association; 2013.

[16] Young RC, Biggs JT, Ziegler VE, Meyer DA. A rating scale for mania: reliability, validity and sensitivity. Br J Psychiatry. 1978;133:429-435. doi:10.1192/bjp.133.5.429 728692

[17] Randolph C, Tierney MC, Mohr E, Chase TN. The Repeatable Battery for the Assessment of Neuropsychological Status (RBANS): preliminary clinical validity. J Clin Exp Neuropsychol. 1998;20(3):310-319. doi:10.1076/jcen.20.3.310.823 9845158

[18] Phillips R, Cheung YB, Collinson SL, . The equivalence and difference between the English and Chinese language versions of the Repeatable Battery for the Assessment of Neuropsychological Status. Clin Neuropsychol. 2015;29(suppl 1):1-18. doi:10.1080/13854046.2015.1034182 25922131

[19] Andrade C. Intent-to-treat (ITT) vs completer or per-protocol analysis in randomized controlled trials. Indian J Psychol Med. 2022;44(4):416-418. doi:10.1177/02537176221101996 35949630 PMC9301744

[20] Fitzgerald PB, Hoy KE, Elliot D, . A pilot study of the comparative efficacy of 100 Hz magnetic seizure therapy and electroconvulsive therapy in persistent depression. Depress Anxiety. 2018;35(5):393-401. doi:10.1002/da.22715 29329499

[21] Kayser S, Bewernick BH, Grubert C, Hadrysiewicz BL, Axmacher N, Schlaepfer TE. Antidepressant effects, of magnetic seizure therapy and electroconvulsive therapy, in treatment-resistant depression. J Psychiatr Res. 2011;45(5):569-576. doi:10.1016/j.jpsychires.2010.09.008 20951997

[22] Kayser S, Bewernick B, Axmacher N, Schlaepfer TE. Magnetic seizure therapy of treatment-resistant depression in a patient with bipolar disorder. J ECT. 2009;25(2):137-140. doi:10.1097/YCT.0b013e31817dc45a 19057399

[23] Noda Y, Daskalakis ZJ, Downar J, Croarkin PE, Fitzgerald PB, Blumberger DM. Magnetic seizure therapy in an adolescent with refractory bipolar depression: a case report. Neuropsychiatr Dis Treat. 2014;10:2049-2055. 25382978 10.2147/NDT.S71056 PMC4222618

[24] Tang VM, Blumberger DM, Dimitrova J, . Magnetic seizure therapy is efficacious and well tolerated for treatment-resistant bipolar depression: an open-label clinical trial. J Psychiatry Neurosci. 2020;45(5):313-321. doi:10.1503/jpn.190098 31922372 PMC7850154

[25] Tang VM, Blumberger DM, Hill AT, . Magnetic seizure therapy for the treatment of suicidality in bipolar depression. Biol Psychiatry. 2021;90(10):e51-e53. doi:10.1016/j.biopsych.2020.09.020 33172609

[26] Levenberg K, Cordner ZA. Bipolar depression: a review of treatment options. Gen Psychiatr. 2022;35(4):e100760. doi:10.1136/gpsych-2022-100760 36035376 PMC9358943

[27] Noda Y, Daskalakis ZJ, Fitzgerald PB, Downar J, Rajji TK, Blumberger DM. Magnetic seizure therapy-induced mania: a report of 2 cases. J ECT. 2015;31(1):e4-e6. doi:10.1097/YCT.0000000000000145 24839980

[28] Wang J, Vila-Rodriguez F, Ge R, . Accelerated magnetic seizure therapy (aMST) for treatment of major depressive disorder: a pilot study. J Affect Disord. 2020;264:215-220. doi:10.1016/j.jad.2019.12.022 32056753

[29] Li J, Zhang X, Jiang J, . Comparison of electroconvulsive therapy and magnetic seizure therapy in schizophrenia: structural changes/neuroplasticity. Psychiatry Res. 2022;312:114523. doi:10.1016/j.psychres.2022.114523 35378453

[30] Cotovio G, Oliveira-Maia AJ. Functional neuroanatomy of mania. Transl Psychiatry. 2022;12(1):29. doi:10.1038/s41398-022-01786-4 35075120 PMC8786958

[31] Deng ZD, Lisanby SH, Peterchev AV. Electric field strength and focality in electroconvulsive therapy and magnetic seizure therapy: a finite element simulation study. J Neural Eng. 2011;8(1):016007. doi:10.1088/1741-2560/8/1/016007 21248385 PMC3903509

[32] Leaver AM, Espinoza R, Wade B, Narr KL. Parsing the network mechanisms of electroconvulsive therapy. Biol Psychiatry. 2022;92(3):193-203. doi:10.1016/j.biopsych.2021.11.016 35120710 PMC9196257

[33] Segi-Nishida E, Warner-Schmidt JL, Duman RS. Electroconvulsive seizure and VEGF increase the proliferation of neural stem-like cells in rat hippocampus. Proc Natl Acad Sci U S A. 2008;105(32):11352-11357. doi:10.1073/pnas.0710858105 18682560 PMC2516270

[34] Thomann PA, Wolf RC, Nolte HM, . Neuromodulation in response to electroconvulsive therapy in schizophrenia and major depression. Brain Stimul. 2017;10(3):637-644. doi:10.1016/j.brs.2017.01.578 28162976

[35] Wang J, Tang Y, Curtin A, . ECT-induced brain plasticity correlates with positive symptom improvement in schizophrenia by voxel-based morphometry analysis of grey matter. Brain Stimul. 2019;12(2):319-328. doi:10.1016/j.brs.2018.11.006 30473477

[36] Weissman CR, Blumberger DM, Dimitrova J, . Magnetic seizure therapy for suicidality in treatment-resistant depression. JAMA Netw Open. 2020;3(8):e207434. doi:10.1001/jamanetworkopen.2020.7434 32809030 PMC7435344

[37] Jiang J, Li Q, Sheng J, . 25 Hz magnetic seizure therapy is feasible but not optimal for Chinese patients with schizophrenia: a case series. Front Psychiatry. 2018;9:224. doi:10.3389/fpsyt.2018.00224 29896130 PMC5986936

[38] Bai S, Loo C, Al Abed A, Dokos S. A computational model of direct brain excitation induced by electroconvulsive therapy: comparison among three conventional electrode placements. Brain Stimul. 2012;5(3):408-421. doi:10.1016/j.brs.2011.07.004 21962983

[39] Deng ZD, Argyelan M, Miller J, . Electroconvulsive therapy, electric field, neuroplasticity, and clinical outcomes. Mol Psychiatry. 2022;27(3):1676-1682. doi:10.1038/s41380-021-01380-y 34853404 PMC9095458

[40] Ousdal OT, Argyelan M, Narr KL, ; GEMRIC. Brain changes induced by electroconvulsive therapy are broadly distributed. Biol Psychiatry. 2020;87(5):451-461. doi:10.1016/j.biopsych.2019.07.010 31561859

