# Supplementary material for: Magnetic Seizure Therapy vs Modified Electroconvulsive Therapy in Patients With Bipolar Mania: A Randomized Clinical Trial
Source: JAMA Netw Open. 2024 Apr 29;7(4):e247919. doi: 10.1001/jamanetworkopen.2024.7919 (PMC11059045; doi:10.1001/jamanetworkopen.2024.7919)
Supplement: Supplement 1. — Trial Protocol [file jamanetwopen-e247919-s001.pdf]

Study Protocol

Title:

Magnetic Seizure Therapy for Bipolar Mania

Version: 02

Date: 11-16-2017

Principal Investigator: Jianhua Sheng

Table of Contents

1. Research Title, Main Funding ....- 3 -
2. Sponsor, Site, Principal Investigator ....- 3 -
3. Study Objectives ....- 3 -
4. Background ....- 3 -
5. Experimental Design: Randomized Controlled Study ....- 6 -
6. Participants..- 6 -
7. Sample Size ....- 7 -
8. MST and ECT Procedures ....- 7 -
9. Outcome Measures ....- 8 -
10. The Study Flow Chat ....- 10 -
11. Exit (Dropout) Criteria ....- 10 -
12. Risk and Its Control..- 11 -
13. Recording, Reporting, and Handling of Adverse Events ....- 12 -
14. Data Management and Statistical Analysis Plan ....- 13 -
15. Quality Control for the Trial ....- 13 -
16. The Planned Progress of the Trial ....- 14 -
17. Reference ....- 15 -

1. Research Title, Main Funding

Title: Magnetic Seizure Therapy for Bipolar Mania

Funding: Science and Technology Commission of Shanghai Municipality (17411969900)

2. Sponsor, Site, Principal Investigator

Sponsor：Shanghai Mental Health Center

Site：Shanghai Mental Health Center, 600 Wanping Nan Road, Shanghai, China

Principal Investigator: Jianhua Sheng

3. Study Objectives

(1) To develop a Magnetic Seizure Therapy (MST) treatment protocol for patients with bipolar mania

(2) To investigate MST induced changes of brain plasticity and cognitive functions in patients with bipolar mania.

(3) To identify biological biomarkers for predicting the MST efficacy and its underlying meachnism

4. Background

4.1 Current treatments for bipolar mania

Bipolar disorder (BD) is characterized by cycling between manic and depressive states. A manic episode, specifically, is a feature of bipolar disorder where an individual experiences heightened mood. The lifetime prevalence of BD is 1% [1], with onset often occurring during adolescence. The resulting years of life lost due to disability rank the eighth among all diseases and the third among mental disorders in low- to middle-income countries [2]. Even during periods of remission, individuals with BD may still experience significant declines in cognitive function and quality of life [3]. Systematic reviews indicate that electroconvulsive therapy (ECT) is a crucial treatment modality for BD, particularly in cases of severe or treatment-resistant conditions [4].

ECT delivers electrical stimulation directly to the brain which induces seizure

activity and achieves therapeutic effects. It is widely used in the treatment of various mental disorders, including mood disorders (major depressive disoder and bipolar disorder) and schizophrenia [5]. However, due to the poor conductivity and significant shunting of the skull, the stimulus intensity applied in ECT is often high and challenging to focus on a specific area. Moreover, it frequently passes through deep brain structures such as the hippocampus and thalamus, leading to noticeable side effects of ECT, particularly cognitive impairments like orientation disorders and memory deficits [6]. In recent years, considering the adverse impact of ECT on patients' neurocognitive function, the U.S. FDA is contemplating categorizing it as a Class 2 (general controls and special controls) or Class 3 (general controls and premarket approval) medical technology [7].

4.2 Development of MST

Since the 1980s, the international community has developed repetitive transcranial magnetic stimulation (rTMS) technology. Leveraging the physics of electromagnetic conversion, this technique initially converts the electrical current into a transient pulsatile magnetic field. This magnetic field can then effortlessly penetrate the skull, reach the cerebral cortex, and generate induced current. TMS is a safe and non-invasive intervention which does not induce seizure. During TMS treatments, patients are awake with no significant discomfort [8]. Later, scientists have developed MST technique that deliberately induce seizures with high-intensity and high-frequency magnetic stimulations around the year 2000 [9].

MST is a novel treatment that induces seizures through the rapid and strong magnetic field. Because the magnetic field easily penetrates the skull, it can selectively stimulate a specific site and only excite the local cortex, inducing seizures by exciting the local cortex. In comparison to ECT, during the process of inducing seizures, MST may not have a direct current passing through deep brain structures (such as the hippocampus and thalamus). Therefore, the side effects of MST may be less than those of ECT [10]. Since 2000, MST has been applied in the clinical treatment of patients with major depressive disorder and bipolar depression. The findings suggest that MST has efficacy comparable to ECT. In addition, the cognitive side effects which are associated with ECT, are less or milder with MST treatment [9]. However, there is currently no research exploring the use of MST as an adjunctive treatment for bipolar manic.

4.3 The Mechnisim of ECT and MST Treatments

Although ECT treatment has a history of more than eighty years, the therapeutic

mechanisms for treating manic episodes are far from clear. Initiating this project provides us with an opportunity to investigate the biological mechanisms of ECT and MST interventions. Evidence in this regard will support the future optimization of ECT and MST treatment techniques.

We propose a hypothesis that MST may improve GABA-mediated cortical inhibition (CI) deficits in patient. ECT may significantly increase the seizure threshold in patients by inducing seizures, thereby strengthening cortical inhibitory function [11]. A typical ECT treatment course usually involves 8-10 sessions within approximately a month. As the number of ECT treatments accumulates, the seizure threshold of patients continues to rise. The required stimulus intensity to induce seizures also gradually increases. In comparison to the initial treatment, the intensity required for seizures in the final treatment session often needs to be increased by 50% to 100%.

The CI function can be assessed using TMS, reflecting the inhibitory GABA-mediated cortical response [12]. Research on CI function in patients with schizophrenia has yielded relatively stable resultsc by cortical silent period (CSP), short-interval cortical inhibition (SICI), and intracortical facilitation (ICF). Previous studies have demonstrated reduced CI function in BD patients, reflecting an inhibitory deficit [13].

CI can also be assessed using magnetic resonance spectroscopy (MRS) to identify changes in in-vivo GABA levels in BD individuals [14,15]. However, there is currently a lack of longitudinal reports on changes in GABA levels in BD patients related to ECT or MST interventions. We believe that relying on TMS-derived cortical inhibition indicators and GABA levels detected through MRS technology will provide reliable evidence for mutual validation.

CI function can also be assessed in vivo by magnetic resonance spectroscopy (MRS), reflecting the GABA levels. Previous MRS studies have suggested altered GABA levels in BD individuals [14,15]. However, there is currently a lack of longitudinal studies reporting changes in GABA levels in BD related to ECT or MST. We hypothesize that CI indices measured by TMS and GABA levels measured by MRS will provide more evidence for the underlying meachnism of ECT and MST treatments.

4.3.2 The MST Effect on Resting-state Brain Network

Functional MRI (fMRI) provides a way to observe the brain fluctuation associated with individual states and information processing. Resting-state fMRI reflects the spontaneous functional activity of the central nervous system in its baseline state, and existing studies support the theory of disrupted cortical-limbic connectivity in patients

with bipolar disorder [17]. Hence, the application of resting-state brain networks is a potential tool to uncover the biological mechanisms of MST treatment in BD, but such study remains rare.

4.4 The Hypothesis of the Present Study

ECT and MST may have comparable efficacy for treating bipolar mania. There may be common mechanism of the efficacy that results from enhenced CI and the plasticity of functional connectivity. However, MST may have less side effects than ECT due to that magnetic stimulations may not pass the deep subcortical regions.

MST has been a novel treatment for MDD, bipolar depression and schizophrenia. However, there is no clinical trial for MST in bipolar mania. The present study aims to conduct a randomized controlled clinical trial to compare MST and ECT efficacy in bipolar mania. We also will expolre the biological mechnism of MST treatments by combining GABA measures and resting-state functional connectivity.

5. Experimental Design: Randomized Controlled Study

Randomization Method: A random number sequence will be generated and recorded by the computer, with each random number corresponding to a specific group (A for ECT group or B for MST group). Patients meeting the inclusion criteria will be assigned to either Group A or Group B based on the random number corresponding to their serial number.

6. Participants

This study plans to recruit 60 patients with bipolar manic for receiving MST and ECT treatments. Additionally, 30 healthy controls will be recruited without any intervention.

6.1 Heatlhy controls

Healthy controls will only participate in baseline cognitive function assessment (rBANS), cortical inhibition (including TMS and MRS detection), and resting-state fMRI assessments. They will not receive MST or MST treatmetns and not particpate in follow-up assessments.

The healthy control will give written consent beofre participanting in the assessments. They will be matched with the patient group in terms of sex, age (between

18 and 55 years), years of education, and socio-economic status. In addtion, they will be screened to exclude an occurrence of psychiatric disorders by the DSM-5 diagnostic criteria. They have no family history of psychiatric disorders, no history of substance dependence, are not currently taking any medication, and do not have any concurrent major physical illnesses.

6.2 Patients with Bipolar Mania

Inclusion criteria: ① meeting the diagnostic criteria by the fifth edition of the Diagnostic and Statistical Manual of Mental Disorders (DSM-5) and currently during a manic episode; ②inpatients in Shanghai Mental Health Center; ③clinical indications of receiving convulsive treatment, such as severe psychomotor excitement or retardation, attempts of suicide, being highly aggressive, pharmacotherapy intolerance, and ineffectiveness of antipsychotics; ④age between 18 and 55 years old ; ⑤moderate to severe manic symptoms with scores of Young Manic Rating Scale (YMRS) ≥10; ⑥Written informed consent.

Exclusion criteria: ①comorbidity with other mental disorders by DSM-5; ②with unremovable metal implants; ③a laboratory abnormality with an impact on the efficacy of treatments or safety; ④severe physical diseases (stroke, heart failure, liver failure, neoplasm, or immune deficiency), a laboratory abnormality with an impact on the efficacy of treatments or safety; ⑤Alcohol or substance dependence in the 30 days prior to the start of the trial or in the 6 months prior to the trial; ⑥failure to respond to an adequate trial of ECT; ⑦being or intending to get pregnant during the study; ⑧ other conditions that investigators consider to be inappropriate to participate in this trial.

7. Sample Size

Due to the lack of preivous MST study as an adjunctive treatment for bipolar mania, we estimated based on previous ECT study. The following frmula was applied with α=0.05, β=0.80,

$$n = 2\pi(1-\pi)\left[\frac{(u_{1-\alpha} + u_{1-\beta})}{\delta}\right]^2$$

The sample size is 30 for ECT and MST group, respectively.

8. MST and ECT Procedures

General anesthesia was administered for each participant before each ECT/MST

session by administering propofol (1.82-2.44mg/kg) and etomidate (0.21-0.3mg/kg). The succinylcholine (1mg/kg) was used for muscle relaxation to prevent fractures and other injuries, and the atropine (0.5mg) was used for reducing respiratory secretions. After each ECT/MST session, patients stayed under observation for half an hour and were not allowed to eat until two hours later.

MST Procedure: Patients completed 2 or 3-session MST on alternate days every week and achieved 8-10 sessions accumulatively within 4 weeks. MST was administered using the MagPro X100 device with a twin coil (Twin Coil-XS, MagVenture A/S, Denmark) placed and centered on the vertex. The intensity was at 100% device output with 75 Hz frequency. The duration of magnetic stimulations was determined by a titration method until a proper seizure (seizure duration ≥ 15 s) was generated. The duration started from 4 s and was increased by 4 s or 8 s up to a maximum of 20 s.

ECT Procedure: Patients completed 2- or 3-session MECT on alternate days weekly and accumulated 8-10 sessions within 4 weeks. ECT was performed using the Thymatron System IV device (Somatics, USA) with electrodes placed over the bilateral temporal cortex. The electrical stimulation wave width was at 1.0 ms. The energy of electrical stimulation was determined according to the patient's (age × 0.8 × 100%). The energy percent was increased by 5% until a proper seizure (seizure duration ≥ 25 s).

EEG Recording: During each MST/ECT session, patch electrodes were placed in the bilateral frontal regions to record electroencephalogram (EEG). The epileptiform discharge times induced by MST/ECT were recorded as the effective seizure duration.

9. Outcome Measures

9.1 Primary outcomes

Designated qualified psychiatrists assessed all patients' symptoms before and after the entire MST and ECT treatments. Manic symptoms were assessed by YMRS[1]. The reduction rate of YMRS total scores was used as the primary outcome. The response is defined as a >50% reduction of YMRS total scores compared to the baseline.

9.2 Secondary outcomes

9.2.1 Other clinical outcomes

Before and after the MST/ECT treatments, depressive symptoms were assessed

using the Montgomery-Asberg Depression Rating Scale (MADRS) and clinical outcome using CGI.

9.2.2 Neurocognitive outcomes

Neurocognitive function were evaluated using the Repeatable Battery for the Assessment of Neuropsychological Status (RBANS) [2]. We used the Chinese version of RBANS, which has good reliability and validity [3], consisting of 12 tasks for five cognitive domains: Immediate Memory (IM), Visuospatial / Constructional (V/C), Language, Attention, and Delayed Memory (DM). A trained staff operated the RBANS assessment. Some participants did not complete the RBANS assessments due to emotional instability and behavioral incooperation in the acute phase. MADRS total scores, RBANS total scores, and scores of each RBANS cognitive domain were secondary outcomes.

9.2.3 CI outcomes

Magnetic stimulation is applied to the left motor cortex through TMS and surface electromyography (EMG) electrodes are placed at the the abductor pollicis brevis muscle position. Motor-evoked potentials (MEPs) are recorded. Multiple CI measueres were obtained.

9.2.4 GABA measures by MRS

Using Siemens 3T Verio magnetic resonance imaging (MRI) system, the MEGA-PRESS sequence is employed with parameters set as follows: TR (repetition time) = 2000ms, TE (echo time) = 69ms, and an average of 128 acquisitions. The data is collected with the region of interest in the frontal lobe, and reconstruction is performed in a plane perpendicular to the splenium of the corpus callosum, with voxel size set at 30mm * 30mm * 30mm.

9.2.5 fMRI measures

Before and after the ECT or MST treatment, MRI data will be collected at the Shanghai Mental Health Center using a Siemens 3T MRI scanner, including T1, T2, and resting-state fMRI data.

For T1-weighted images, the magnetization-prepared rapid gradient-echo (MP-RAGE) sequence is employed with a repetition time (TR) of 1800ms, echo time (TE) of 3.4ms, inversion time (TI) of 1100ms, flip angle (FA) of 7 degrees, field of view (FOV) of 256mm, voxel size of 1mm*1mm*1mm, and axial continuous scanning of 176 layers. The GRAPPA algorithm is used with a parallel imaging factor of 2, and the

entire scanning process lasts for 3 minutes and 57 seconds.

For T2-weighted images, the sequence has a repetition time (TR) of 3200ms, echo time (TE) of 224ms, FOV of 256mm, voxel size of 1mm*1mm*1mm, and axial continuous scanning of 176 layers. The GRAPPA algorithm with a parallel imaging factor of 2 is applied, and the scanning process takes 4 minutes and 43 seconds.

For resting-state functional images, the sequence has a repetition time (TR) of 6000ms, echo time (TE) of 30ms, flip angle (FA) of 90 degrees, FOV of 192mm, 60 measurements, voxel size of 2mm*2mm*2mm, and axial continuous scanning of 67 layers. The entire scanning process for resting-state functional images lasts for 6 minutes and 32 seconds. During scanning, all participants are instructed to keep their eyes open without any specific thoughts.

10. The Study Flow Chat

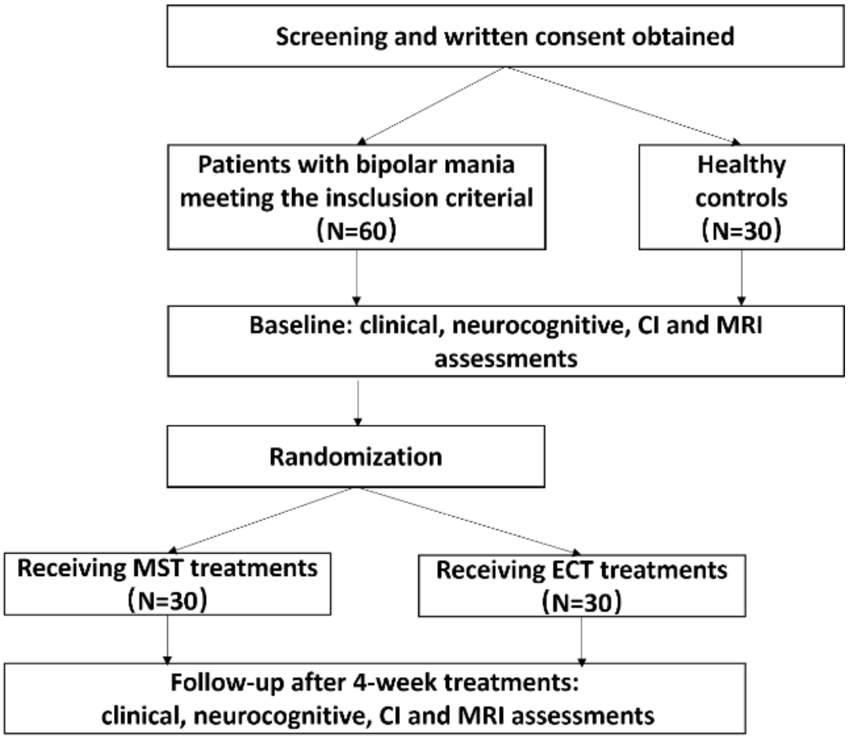

Figure 1. The study flow chat

11. Exit (Dropout) Criteria

During the trial, if the following conditions occur, the researcher is responsible for terminating the subject's continued participation in the trial (excluding them).

Subsequently, a detailed explanation and assessment of the termination reason, date, and the clinical process leading to the event should be recorded on the case report form. Subjects are free to withdraw (dropout).

(1) As determined by the investigator, the trial cannot continue due to adverse events or abnormal laboratory test values; (2) The subject want to withdraw from the trial; (3) Significant protocol violations (including those deemed non-compliant by the investigator); (4) Other situations where the investigator deems it inappropriate or difficult to continue the trial.

Subjects have the right to withdraw from the trial at any time without providing any reason. If a subject decides to withdraw from the trial, the researcher should inquire about the reasons to the extent possible and document them in the source documents and case report forms. When a subject withdraws from the trial prematurely, an exit assessment must be conducted.

12. Risk and Its Control

Although there may be risks and adverse reactions during the process of ECT/MST treatment, the adverse reactions are relatively few and mild, and serious complications are rare. Possible accidents and adverse reactions include: (1) Individual patients may be insensitive to muscle relaxants, leading to accidents such as fractures and joint dislocations; (2) Some patients may experience reversible headaches, nausea, muscle pain, and recent memory disturbances in the short term. Anesthetic accidents and complications may include: (1) Vomiting, aspiration, and laryngospasm may occur during anesthesia treatment; (2) Patients may be allergic to anesthesia drugs; (3) Due to individual differences, patients may experience slow metabolism of anesthetic drugs, leading to delayed recovery of spontaneous breathing or even requiring tracheal intubation; (4) Anesthesia may induce and exacerbate pre-existing diseases, potentially causing disruptions in heart, brain, and respiratory functions.

We will implement several measures to reduce these accidents and adverse reactions, such as: (1) Strict fasting within 6 hours before the procedure, and no water intake within 3 hours before the procedure; (2) Removal of dentures and all kinds of accessories before the procedure; (3) After the procedure, the patient must remain in the treatment room until fully awake and obtain permission from the doctor to leave. A healthcare professional or a guardian responsible for the patient's medical care must accompany the patient; (4) Eating is allowed 2 hours after the procedure; (5) Within 12

hours after the end of general anesthesia, activities such as climbing, driving, and making important decisions should be avoided.

13. Recording, Reporting, and Handling of Adverse Events

If any clinically significant findings are discovered in laboratory tests, vital signs, electrocardiograms, and other examinations, the investigator or assistant researcher should record them on the "Pre-existing Conditions and Adverse Events" page of the case report form. For findings that have clinical significance and can lead to a specific diagnosis, the diagnosis should be documented as an adverse event. If a diagnosis cannot be made, the clinical significance should be determined by the investigator, and findings deemed to have clinical significance should be recorded as adverse events.

After the investigator discovers a serious adverse event, they must report it to the designated personnel within 24 hours through a method approved by the Principal Investigator (PI). Following a telephone report, the investigator must promptly complete and send the study-specific Serious Adverse Event Record Form for the following events: death; initiation of hospitalization or prolongation of hospital stay; life-threatening situations (immediate risk of death); persistent or significant disability or functional impairment; congenital anomalies or birth defects; any event that the investigator deems significant for any reason.

Accurately complete the Adverse Event Record Form during the trial, documenting the occurrence time, severity, duration, measures taken, and outcome of adverse events.

In the event of a serious adverse event, immediate necessary measures must be taken to ensure the safety of the subjects. All adverse events should be thoroughly investigated, with detailed documentation of the process and outcomes of the intervention, until a satisfactory resolution or stabilization of the condition is achieved. In the case of laboratory abnormalities, tracking should continue until normalization. Follow-up methods can be chosen based on the severity of the adverse reaction and may include hospitalization, outpatient visits, home visits, telephone follow-ups, correspondence, or other forms.

Any serious adverse reaction events occurring during the trial must be reported immediately to the local or principal medical ethics committee and the sponsor. The "Serious Adverse Event Report Form" should be completed. Notify the units and contacts listed in the informed consent form. Follow-up for unresolved adverse events:

All adverse events should be tracked until a satisfactory resolution or stabilization of the condition is achieved.

14. Data Management and Statistical Analysis Plan

14.1 Data Management

The case report forms are uniformly organized and kept by dedicated personnel. They are only taken out when data entry personnel are inputting and verifying data, and are promptly returned to their original state after completing these tasks. During the project implementation, a dedicated database is established to track various information about the study subjects and record adverse events. A dedicated person is responsible for the security management of the database and utilizing it for research quality control.

14.2 Statistical Analysis Plan

We will use SPSS for statistical analysis, employing statistical methods such as t-tests, analysis of variance, and correlation analysis based on the data types and distribution characteristics. For the MRS data, LC-Model will be utilized. After preprocessing, resting-state fMRI data will be analyzed using software packages such as REST and GIFT.

(1) Baseline coparisons: To compare the differences in neurocognitive function, CI indicators, and resting-state fMRI measures between the MST group, ECT group and healthy control group; To explore the correlation between CI indicators and GABA levels detected within each group.

(2) Longitudinal comparisons: Compare the longitudinal changes of clinical, neurocogntive and CI measures before and after ECT and MST interventions.

(3) fMRI: the medial prefrontal cortex within the defalut mode network (DMN) will be chosen as a seed to calcualte its functional connectivities the posterior cingulate gyrus, precuneus and the bilateral inferior parietal lobules. We hypothesize that patients with bipolar mania may show enhanced connectivity within the DMN, and this connectivity will be improved after MST or ECT intervention.

15. Quality Control for the Trial

We will take the following steps to perform quality control for the clinical trial:

We establish a dedicated research team for this trial with Dr Jianhua Sheng as the

PI. We will establish SOP for the present study.

A trained staff will regularly conduct monitoring throughout the trial. The staff will examine whether the clinical facilities at the research centers meet the requirements, assure that research personnel adhere to the protocol and accurately record trial results. During each monitoring visit, the staff must also review subject data. Monitors are also responsible for ensuring that every participant in the study has signed the informed consent form.

The research team consists of staffs who have received GPC training and work under the guidance of senior professionals.

The laboratory establishs SOP and QC procedures for the experimental observation indicators.

Throughout the trial, the research team members hold regular weekly project coordination meetings to discuss the progress of the project and plan for the next steps.

16. The Planned Progress of the Trial

2017	**July- September：** Preparation and startup for the trial.<br>**October- December：** Collect data from 6 patients for intervention assessment and simultaneously collect data from 3 healthy controls.
2018	**January-March：** Collect data from 6 patients for intervention assessment and simultaneously collect data from 3 healthy controls.<br>**April-June：** Collect data from 6 patients for intervention assessment and simultaneously collect data from 3 healthy controls.<br>**July- September：** Collect data from 6 patients for intervention assessment and simultaneously collect data from 3 healthy controls.<br>**October- December：** Collect data from 6 patients for intervention assessment and simultaneously collect data from 3 healthy controls.
2019	**January-March：** Collect data from 6 patients for intervention assessment and simultaneously collect data from 3 healthy controls.<br>**April-June：** Collect data from 6 patients for intervention assessment and simultaneously collect data from 3 healthy controls.<br>**July- September：** Collect data from 6 patients for intervention assessment and simultaneously collect data from 3 healthy controls.<br>**October- December：** Collect data from 6 patients for intervention assessment and simultaneously collect data from 3 healthy controls.

2020	**January-March：** Collect data from 6 patients for intervention assessment and simultaneously collect data from 3 healthy controls.<br>**April-June：Data analysis**

17. Reference

[1] Merikangas KR, Jin R, He JP, Kessler RC, Lee S, Sampson NA, et al. Prevalence and correlates of bipolar spectrum disorder in the world mental health survey initiative. Archives of General Psychiatry. 2011;68(3):241-51.

[2] Mathers C, Fat D M, Boerma J T. The global burden of disease: 2004 update. World Health Organization, 2004.

[3] Xiao L, Gao Y, Zhang L, Chen P, Sun X. The relationship between cognitive function and quality of life in euthymic Chinese patients with bipolar disorder. Psychiatry Research. 2016; 246: 427-431.

[4] Versiani, Marcio, Elie Cheniaux, and J. Landeira-Fernandez. Efficacy and safety of electroconvulsive therapy in the treatment of bipolar disorder: a systematic review. The journal of ECT. 2011; 27 (2): 153-164.

[5] Leiknes K A, Schweder L J, Høie B. Contemporary use and practice of electroconvulsive therapy worldwide. Brain and behavior. 2012; 2(3): 283-344.

[6] Group UER. Efficacy and safety of electroconvulsive therapy in depressive disorders: a systematic review and meta-analysis. Lancet. 2003; 361(9360):799-808.

[7] Allan CL, Ebmeier KP. The use of ECT and MST in treating depression. Int Rev Psychiatry. 2011; 23(5):400-12.

[8] Wang J，Yang XM，Li H. Physical therapy for mental disorders (in Chinese). ISBN: 978-7-117-16449-8. People's Medical Publishing House 2012.

[9] Chen Z, Jiang JL, Jia YP, Wang J, Li CB. Basic and clinical research progress of magnetic seizure therapy (in Chinese). Mental health in Sichuan. 2016; 29(5): 482-485.

[10] Zyss T, Zieba A, Hese RT, Dudek D, Grabski B, Gorczyca P, Modrzejewska R. Magnetic seizure therapy (MST)--a safer method for evoking seizure activity than current therapy with a confirmed antidepressant efficacy. Neuro Endocrinol Lett. 2010; 31(4):425-37.

[11] Farzan F, Boutros NN, Blumberger DM, Daskalakis ZJ. What Does the Electroencephalogram Tell Us About the Mechanisms of Action of ECT in Major

Depressive Disorders? J ECT. 2014; 30(2):98-106.

[12] Wang J. New Treatment Technologies for Mental Disorders - Transcranial Magnetic Stimulation (in Chinese). Internal Medicine Theory and Practice. 2011; 6(3): 171-175.

[13] Ruiz-Veguilla M, Martín-Rodríguez JF, Palomar FJ, Porcacchia P, Álvarez de Toledo P, Perona-Garcelán S, et al. Trait-and state-dependent cortical inhibitory deficits in bipolar disorder. Bipolar disorders. 2016; 18 (3): 261-271.

[14] Atagün Mİ, Şıkoğlu EM, Soykan Ç, Serdar Süleyman C, Ulusoy-Kaymak S , Çayköylü A, et al. Perisylvian GABA levels in schizophrenia and bipolar disorder. Neuroscience Letters. 2017; 637:70-74.

[15] Brady RO Jr, McCarthy JM, Prescot AP, Jensen JE, Cooper AJ, Cohen BM, et al. Brain gamma -aminobutyric acid (GABA) abnormalities in bipolar disorder. Bipolar Disorders. 2013; 15(4):434-439.

[16] Vargas C, López-Jaramillo C, Vieta E. A systematic literature review of resting state network—functional MRI in bipolar disorder. Journal of affective disorders. 2013; 150(3): 727-735.
