# Supplementary material for: Magnetic Seizure Therapy vs Modified Electroconvulsive Therapy in Patients With Bipolar Mania: A Randomized Clinical Trial
Source: JAMA Netw Open. 2024 Apr 29;7(4):e247919. doi: 10.1001/jamanetworkopen.2024.7919 (PMC11059045; doi:10.1001/jamanetworkopen.2024.7919)
Supplement: Supplement 2. — eMethods 1. Sample Size Calculation eMethods 2. Results of Per-Protocol Analysis eReference eTable 1. Demographic and Clinical Characteristics of Patients Before Receiving Modified Electroconvulsive Therapy (ECT) and Magnetic Seizure Therapy (MST) eTable 2. Clinical Characteristics of Patients Before and After Receiving Magnetic Seizure Therapy (MST) and Modified Electroconvulsive Therapy (ECT) eTable 3. Neurocognitive Assessments of Patients Before and After Receiving Magnetic Seizure Therapy (MST) and Modified Electroconvulsive Therapy (ECT) [file jamanetwopen-e247919-s002.pdf]

Supplemental Online Content

Chen S, Sheng J, Yang F, et al. Magnetic seizure therapy vs modified electroconvulsive therapy in patients with bipolar mania: a randomized clinical trial. *JAMA Netw Open*. 2024;7(4):e247919. doi:10.1001/jamanetworkopen.2024.7919

eMethods 1. Sample Size Calculation

eMethods 2. Results of Per-Protocol Analysis

eReference

eTable 1. Demographic and Clinical Characteristics of Patients Before Receiving Modified Electroconvulsive Therapy (ECT) and Magnetic Seizure Therapy (MST)

eTable 2. Clinical Characteristics of Patients Before and After Receiving Magnetic Seizure Therapy (MST) and Modified Electroconvulsive Therapy (ECT)

eTable 3. Neurocognitive Assessments of Patients Before and After Receiving Magnetic Seizure Therapy (MST) and Modified Electroconvulsive Therapy (ECT)

This supplemental material has been provided by the authors to give readers additional information about their work.

© 2024 Chen S et al. *JAMA Network Open.*

eMethods 1. Sample Size Calculation

The present trial is a non-inferiority design base on the hypothesis of the primary outcome. We hypothesized that the primary outcome (the reduction rate of the total Young Manic Rating Scale [YMRS] score and response rate) would be comparable between groups receiving electroconvulsive therapy (ECT) and magnetic seizure therapy (MST) groups. When we designed this study, there had been no MST study in patients with bipolar mania yet. Thus, we estimated sample size according to the previous MST study by Kayser et al. (2015) [1], in which 26 patients suffering from treatment-resistant depression underwent MST. Considering ~15% dropout rate, we determined a sample size of 30 for each group (MST or ECT), respectively.

eMethods 2. Results of Per-Protocol Analysis

We further performed a per-protocol (PP) analysis, which included 20 patients in the MST group and 18 in the ECT group. Patients included in the PP analysis completed at least 8-session treatments.

Demographic and clinical characteristics

The age, sex, years of education and marriage status were matched between MST and ECT groups (all P>0.05, eTable 1). The daily chlorpromazine-equivalent doses of atypical antipsychotics in the MST group were significantly higher than those in the ECT group at baseline (Z=-2.701, p=0.007) and comparable after the treatments (Z=-0.352, p=0.725). During treatments, the increment of the daily chlorpromazine-equivalent doses in the ECT group was significantly higher than in the MST group (Z=-2.699, p=0.007). The MARDS score, YMRS total score, and scores of each YMRS item were comparable between the ECT and MST groups at baseline (all p>0.05).

Clinical outcomes

Seventeen of 20 patients (85.0%) responded to MST treatments, while 17 of 18 patients (94.4%) responded to ECT treatments. There was no significant difference in the response rate between these two groups (χ^2=0.344, p= 0.606). Either the reduction of YMRS total scores (MST: 29.95±11.5; ECT: 30.78±10.1; t=0.235, p=0.816) or the

© 2024 Chen S et al. *JAMA Network Open.*

YMRS reduction rate (MST: 0.80±0.23; ECT: 0.86±0.15; Z=-0.950, p=0.342) did not show any significant between-group difference.

The repeated-measures ANOVA analysis showed significant main effects of time on the MARDS score, YMRS total score, and scores of each YMRS item (eTable 2, all p<0.05). The YMRS total and 11 YMRS item scores significantly decreased after the ECT and MST treatments. There were no significant between-group effects or significant interactions of time × group (all p>0.05).

Neurocognitive outcomes

Fourteen patients in the ECT group and 14 in the MST group completed the RBANS neurocognitive assessments at baseline and after treatments. For RBANS total scores, there were no significant main effects of time (F(1,26)=0.092, p=0.764) or group (F(1,26)=1.164, p=0.291) or interaction of time × group (F(1,26)=0.181, p=0.674).

For the RBANS language domain, there was a significant time × group interaction (F(1,26)=7.602, p=0.011) and a main effect of group (F(1,26)=10.382, p=0.003). We performed the post-hoc analysis using Wilcoxon Signed Ranks Test and found that patients receiving ECT treatments had worse language performances after the treatments than before (z=-2.003, p=0.045), but patients receiving MST treatments showed a trend-level improvement of language performances (z=-1.754, p=0.080).

For the RBNAS attention domain, there was a significant main effect of time (F (1,26) =4.639, p=0.041). Patients in the ECT group showed a trend-level increase in attention scores after treatments (t=-1.863, p=0.085), while patients in the MST group (t=-1.155, p=0.269) showed no significant changes after treatments.

For the other domains (IM, V/C, and DM), there was no significant main effect of time, the main effect of group, or interaction of time × group (all p>0.05).

Seizure durations

The mean seizure duration in the MST group (10.85±3.6 s) was significantly longer than that in the ECT group (36.53±10.0 s; Z=-5.263, p<0.001). The seizure duration

© 2024 Chen S et al. *JAMA Network Open.*

was negatively correlated with the changes in language scores in the MST group (r=-0.668, p=0.009).

Safety, tolerability, and adverse effects

No serious adverse effects were reported in any group. Four patients (2 in the MST group and 2 in the ECT group) reported transient general anesthesia reactions, such as headache and nausea, after treatment and were soon relieved after treatment. One patient in the MST group reported a temporary headache after 6-session treatment and dropped out. No patients in the ECT group dropped out due to physical discomfort during treatments.

eReference

1. Kayser S, Bewernick BH, Matusch A, Hurlemann R, Soehle M, Schlaepfer TE. Magnetic seizure therapy in treatment-resistant depression: clinical, neuropsychological and metabolic effects. *Psychological medicine.* 2015;45(5):1073-1092.

© 2024 Chen S et al. *JAMA Network Open.*

eTable 1. Demographic and Clinical Characteristics of Patients Before Receiving Modified Electroconvulsive Therapy (ECT) and Magnetic Seizure Therapy (MST)

	MST Group n=20	ECT Group n=18	Statistic χ^2/F/t/Z	P value
Sex (M/F)	14/6	11/7	x^2=0.333	0.734
Age (years)	34.8±10.0	32.1±8.7	t=-0.879	0.385
Years of education	13.4±3.8	15.2±3.5	Z=-1.629	0.103
Marriage status (single/married)	13/7	10/8	x^2=0.354	0.741
disease duration (months)	159.7±114.8	98.1±92.5	Z=-1.841	0.073
Inheritance（Y/N）	3/17	7/11	x^2=2.788	0.144
daily chlorpromazine-equivalent doses(mg)	623.3±293.9	376.8±252.5	Z=-2.701	**0.007**
Mood stabilizers （Y/N）	14/6	14/4	x^2=0.296	0.719
Benzodiazepines （Y/N）	4/16	3/15	x^2=0.070	1.000
YMRS	37.2±8.0	35.3±8.3	t=-0.705	0.486
Elevated Mood	3.5±0.6	3.4±0.5	Z=-0.467	0.641
Increased Motor Activity-Energy	3.6±0.5	3.3±0.6	Z=-1.454	0.146
Sexual Interest	2.4±1.2	1.8±1.4	Z=-1.449	0.147
Sleep	3.0±0.6	2.8±0.4	Z=-0.626	0.531
Irritability	4.9±1.9	4.7±2.1	Z=-0.209	0.835
Speech-Rate and Amount increase	5.3±1.6	5.4±1.1	Z=-0.315	0.753
Language-Thought Disorder	2.6±1.1	2.7±1.0	Z=-0.062	0.951
Content of thinking	4.1±2.8	4.4±1.9	Z=-0.207	0.836
Disruptive-Aggressive Behavior	3.7±2.3	3.3±2.3	t=-0.421	0.676
Appearance	1.9±1.3	1.6±1.0	Z=-1.013	0.311
insight	2.5±1.5	2.1±1.4	Z=-1.039	0.299
MARDS	5.9±4.5	7.5±7.1	Z=-0.338	0.736

YMRS, the Young manic rating scale; MARDS, Montgomery- Åsberg Depression Rating Scale; [*], P<0.05

© 2024 Chen S et al. *JAMA Network Open.*

eTable 2. Clinical Characteristics of Patients Before and After Receiving Magnetic Seizure Therapy (MST) and Modified Electroconvulsive Therapy (ECT)

Characteristic	MST Group (n=20)		ECT Group (n=18)		Time		Group		Time × Group	
	Pre-MST	Post-MST	Pre-ECT	Post-ECT	F	P	F	P	F	P
YMRS	37.2±8.0	7.3±8.5	35.3±8.3	4.6±4.2	296.1	<0.001**	1.833	0.184	0.055	0.816
Elevated Mood	3.5±0.6	0.9±1.1	3.4±0.5	0.6±0.6	398.0	<0.001**	0.753	0.391	0.734	0.397
Increased Motor Activity-Energy	3.6±0.5	0.8±1.0	3.3±0.6	0.5±0.6	298.9	<0.001**	2.965	0.094	0.008	0.931
Sexual Interest	2.4±1.2	0.4±0.7	1.8±1.4	0.2±0.5	71.4	<0.001**	2,234	0.144	0.828	0.369
Sleep	3.0±0.6	0.4±0.7	2.8±0.4	0.3±0.5	350.1	<0.001**	1.062	0.310	0.000	0.984
Irritability	4.9±1.9	0.7±1.1	4.7±2.1	0.2±0.5	169.3	<0.001**	0.652	0.425	0.138	0.712
Speech-Rate Amount increase	5.3±1.6	1.0±1.4	5.4±1.1	0.6±0.9	252.4	<0.001**	0.345	0.561	0.861	0.360
Language-Thought Disorder	2.6±1.1	0.4±0.9	2.7±1.0	0.4±0.6	138.1	<0.001**	0.191	0.665	0.003	0.953
Content of thinking	4.1±2.8	0.7±1.5	4.4±1.9	0.3±0.6	92.4	<0.001**	0.007	0.933	0.949	0.337
Disruptive-Aggressive Behavior	3.7±2.3	0.3±1.1	3.3±2.3	0.1±0.2	74.1	<0.001**	0.460	0.502	0.009	0.926
Appearance	1.9±1.3	0.2±0.5	1.6±1.0	0.2±0.4	67.1	<0.001**	0.819	0.371	0.681	0.415
Insight	2.5±1.5	1.6±1.1	2.1±1.4	1.3±1.0	8.093	0.007*	1.498	0.229	0.016	0.900
MARDS	5.9±4.5	1.3±2.9	7.5±7.1	0.9±1.3	47.6	<0.001**	0.292	0.592	1.378	0.248

© 2024 Chen S et al. *JAMA Network Open.*

eTable 3 Neurocognitive Assessments of Patients Before and After Receiving Magnetic Seizure Therapy (MST) and Modified Electroconvulsive Therapy (ECT)

Characteristic	MST Group (n=14)		ECT Group (n=14)		Time		Group		Time × Group	
	Pre-MST	Post-MST	Pre-ECT	Post-ECT	F	P	F	P	F	P
RBANS	82.6±13.8	84.8±14.4	78.8±16.7	78.4±13.9	0.092	0.764	1.164	0.291	0.181	0.674
IM	79.0±13.7	79.0±13.7	77.6±12.4	82.1±19.3	0.485	0.492	0.033	0.857	0.485	0.492
V/C	91.8±19.1	90.7±19.8	88.2±15.0	94.2±11.9	0.790	0.382	0.000	0.995	1.627	0.213
Language	91.9±10.6	97.9±9.9	87.4±16.6	77.5±12.9	0.484	0.493	**10.382**	**0.003***	**7.602**	**0.011***
Attention	94.4±14.3	98.0±16.8	86.5±19.2	92.8±14.7	**4.639**	**0.041***	1.305	0.264	0.352	0.558
DM	77.6±19.8	77.4±17.3	78.1±19.6	70.4±17.9	0.766	0.389	0.343	0.563	0.686	0.415

IM, Immediate Memory; V/C, Visual-spatial / Constructional; DM, Delayed Memory.

© 2024 Chen S et al. *JAMA Network Open.*
