# Supplementary material for: Magnetic Seizure Therapy vs Modified Electroconvulsive Therapy in Patients With Bipolar Mania: A Randomized Clinical Trial
Source: JAMA Netw Open. 2024 Apr 29;7(4):e247919. doi: 10.1001/jamanetworkopen.2024.7919 (PMC11059045; doi:10.1001/jamanetworkopen.2024.7919)
Supplement: Supplement 3. — Data Sharing Statement [file jamanetwopen-e247919-s003.pdf]

Data Sharing Statement

Chen. Magnetic Seizure Therapy vs Modified Electroconvulsive Therapy in Patients With Bipolar Mania. *JAMA Netw Open*. Published April 29, 2024.
doi:10.1001/jamanetworkopen.2024.7919

Data
Data available: No

Additional Information
Explanation for why data not available: The di-identified data could be required by contacting the corresponding author via email.
